# Utilisation of drugs with pharmacogenetic recommendations in children in Switzerland

**DOI:** 10.1038/s41397-025-00378-x

**Published:** 2025-07-11

**Authors:** Nina L. Wittwer, Christoph R. Meier, Carola A. Huber, Romy Tilen, Canan Yilmaz, Henriette E. Meyer zu Schwabedissen, Samuel Allemann, Cornelia Schneider

**Affiliations:** 1 Basel Pharmacoepidemiology Unit, Division of Clinical Pharmacy and Epidemiology, Department of Pharmaceutical Sciences, University of Basel, Basel, Switzerland; 2 Hospital Pharmacy, University Hospital Basel, Basel, Switzerland; 3 Boston Collaborative Drug Surveillance Program, Lexington, MA USA; 4 Department of Health Sciences, Helsana Group, Zürich, Switzerland; 5 Biopharmacy, Department of Pharmaceutical Sciences, University of Basel, Basel, Switzerland; 6 University Children’s Hospital Zurich, Department of Infectious Diseases and Hospital Epidemiology, Zürich, Switzerland; 7 Pharmaceutical Care, Department of Pharmaceutical Sciences, University of Basel, Basel, Switzerland

**Keywords:** Drug safety, Pharmacogenomics, Pharmacogenetics, Public health

## Abstract

Pharmacogenetics (PGx) is increasingly implemented in the adult population, but its potential in children remains uncertain. The aim of this study was to investigate PGx drug utilization in children in Switzerland, using Helsana claims data between 2017 and 2021. We identified 82 drugs with paediatric guideline annotations associated with variants in 24 genes from the Pharmacogenomics Knowledgebase. Of 159 172 children continuously insured, 66.1% claimed at least one PGx drug during the study period. The three PGx drugs with the highest user numbers were systemically administered ibuprofen (59.1%), ondansetron (8.3%), and locally administered fluorouracil (7.5%). Over 96% of all potential drug-gene interactions were caused by seven genes (CYP2C9, CYP2D6, DPYD, CYP2C19, MT-RNR1, CACNA1S, and RYR1). The high number of children claiming PGx drugs in Switzerland implies that a significant number of children could benefit from PGx testing.

## Introduction

Drug-gene-interactions (DGIs) refer to situations where a genetic variant and a drug interact, resulting in an altered drug response [1–3]. Individual genetic factors affecting pharmacodynamics or pharmacokinetics in DGIs can lead to variability in drug exposure and /or response resulting in treatment failure or toxicity [1, 2]. Pharmacogenomics aims to enhance an individuals’ drug response through a personalised, and therefore safer and more effective therapy [4].

Children are a vulnerable patient group and frequently affected by adverse drug reactions [5]. In adults, pharmacogenetic (PGx) testing has been demonstrated to reduce the number of adverse drug reactions, which is associated with fewer hospital admissions due to adverse drug reactions and with improved treatment responses [6–10], but these results cannot easily be transferred to children. Children represent a heterogeneous group ranging from preterm new-borns to adolescents. In the postnatal phase, both age and genotype affect enzyme expression and activity [11]. Moreover, ontogenesis influences the activity of drug-metabolizing enzymes and transporters [12]. Of particular significance in this context are individuals below the age of two years, as ontogenetic variability is most pronounced in this age group [12].

The implementation of PGx for children has proceeded slower than anticipated and is still limited [13, 14]. PGx dosing guidelines applicable for children by the Clinical Pharmacogenetics Implementation Consortium (CPIC) [15, 16], the Dutch Pharmacogenetics Working Group (DPWG) [17, 18], and the Canadian Pharmacogenomics Network for Drug Safety (CPNDS) [19, 20] are available on the Pharmacogenomics Knowledgebase (PharmGKB, www.pharmgkb.org) [21]. These guidelines shall facilitate the implementation of PGx in clinical practice by helping paediatricians and pharmacists to interpret PGx findings.

The majority of existing PGx studies in children focus on the hospital setting and /or specific medical conditions such as mental illnesses, sickle cell anaemia, or children with burns and surgery [22–27]. To assess the benefit of PGx testing in children, it is crucial to understand the utilisation of PGx drugs in paediatrics. To date, most studies examining the prevalence of PGx drugs in children have been conducted in the United States of America (USA) and Canada [23, 28–32]. Currently, there is insufficient research on the frequency of PGx prescriptions in paediatrics in Europe [33].

Therefore, the aim of this study was to analyse the utilisation of PGx drugs in children and adolescents in Switzerland. The objectives were to assess the prevalence of PGx drug prescriptions, to identify the most frequently used PGx drugs, and thereby to determine the most relevant PGx genes in different age groups.

## Materials and methods

### Data source

We used claims data from one of the largest Swiss health insurance providers (Helsana Group), which covers approximately 1.2 million people (15% of the Swiss population) annually across all 26 cantons with basic health insurance. The Helsana database is representative of the Swiss population. This database has previously been used for several drug safety and drug utilisation studies [34–37]. The dataset included demographic information of insured individuals, such as canton of residence, year of birth, and sex. Additionally, it contained records of both therapeutic treatments and diagnostic evaluations carried out in the outpatient setting. Drug claims were recorded using the Anatomical Therapeutical Chemical (ATC) classification. Information on lifestyle factors or over the counter (OTC) drug sales was not available. In our analyses we included claims from children under 18 years of age who were continuously insured from the 1st of January 2017 to the 31st of December 2021. For supplementary analyses of individual 1-year periods, we included claims from children under 18 years of age who were at least insured during the respective year of the study period.

### PGx drugs

PGx drugs were defined as drugs with a dosing or testing recommendation applicable to children on the PharmGKB in April 2023 [21]. We identified 82 drugs as PGx drugs associated with variants in 24 genes (Table 1). We differentiated local vs. systemic applications using the ATC code for the following 12 drugs: amikacin, dapsone, doxepin, fluorouracil, flurbiprofen, gentamicin, ibuprofen, kanamycin, meloxicam, piroxicam, tacrolimus, and tobramycin. If an ATC code included both systemic and local application, we included it in the systemically administered category. We excluded 41 drugs due to lack of guideline recommendations (*n* = 40) or lack of ATC code (toluidine blue). Additionally, the PGx drug claims were categorised into different anatomical groups, using the first level of the ATC code.

**Table 1:** PGx drugs with corresponding genes.

Gene	Gene name	Drug
ABCG2	ATP binding cassette subfamily G member 2	Allopurinol, rosuvastatin
CACNA1S	Calcium voltage-gated channel subunit alpha1 S	Desflurane, enflurane, halothane, isoflurane, methoxyflurane, sevoflurane, succinylcholine
CFTR	Cystic fibrosis transmembrane conductance regulator	Ivacaftor
CYP2B6	Cytochrome P450 2B6	Efavirenz, sertraline
CYP2C19	Cytochrome P450 2C19	Amitriptyline, citalopram, clomipramine, clopidogrel, dexlansoprazole, doxepin, escitalopram, imipramine, lansoprazole, omeprazole, pantoprazole, sertraline, trimipramine, voriconazole
CYP2C9	Cytochrome P450 2C9	Acenocoumarol, celecoxib, flurbiprofen, fluvastatin, fosphenytoin, ibuprofen, lornoxicam, meloxicam, phenytoin, piroxicam, tenoxicam, warfarin
CYP2D6	Cytochrome P450 2D6	Amitriptyline, atomoxetine, clomipramine, codeine, desipramine, doxepin, fluvoxamine, hydrocodone, imipramine, nortriptyline, ondansetron, paroxetine, pimozide, risperidone, tramadol, trimipramine, tropisetron, venlafaxine, vortioxetine
CYP3A4	Cytochrome P450 3A4	Tacrolimus
CYP3A5	Cytochrome P450 3A5	Tacrolimus
CYP4F2	Cytochrome P450 4F2	Warfarin
DPYD	Dihydropyrimidine dehydrogenase	Capecitabine, fluorouracil
G6PD	Glucose-6-phosphate dehydrogenase	Dapsone, methylene blue, nitrofurantoin, pegloticase, primaquine, rasburicase, tafenoquine
HLA-A	Human leukocyte antigen A	Carbamazepine
HLA-B	Human leukocyte antigen B	Abacavir, allopurinol, carbamazepine, fosphenytoin, phenytoin
MT-RNR1	Mitochondrially encoded 12S RNA	Amikacin, gentamicin, kanamycin, paromomycin, plazomicin, streptomycin, tobramycin
NUDT15	Nudix hydrolase 15	Azathioprine, mercaptopurine, thioguanine
RARG	Retinoic acid receptor gamma	Daunorubicin, doxorubicin
RYR1	Ryanodine receptor 1	Desflurane, enflurane, halothane, isoflurane, methoxyflurane, sevoflurane, succinylcholine
SLC28A3	Solute carrier family 8 member A3	Daunorubicin, doxorubicin
SLCO1B1	Solute carrier organic anion transporter family member 1B1	Atorvastatin, fluvastatin, lovastatin, pitavastatin, pravastatin, rosuvastatin, simvastatin
TPMT	Thiopurine S-methyltransferase	Azathioprine, cisplatin, mercaptopurine, thioguanine
UGT1A1	UDP glucuronosyltransferase 1 family, polypeptide A1	Atazanavir
UGT1A6	UDP glucuronosyltransferase 1 family, polypeptide A6	Daunorubicin, doxorubicin
VKORC1	Vitamin K epoxide reductase complex subunit 1	Acenocoumarol, warfarin

*PGx* pharmacogenetic.

### Age groups

Children’s ages were calculated at the end of the calendar year. Children were stratified into five age groups adapted from the Eunice Kennedy Shriver National Institute of Child Health and Human Development: infants (<1 year old), toddlers (1 year old), early childhood (2–4 years old), late childhood (5–10 years old), and adolescence (11–17 years old) [38]. Children can change the age group during the study period.

### Statistical analysis

We performed a retrospective and descriptive analysis and assessed PGx drug claims in all registered children, stratified by age groups. Counts and percentages for drug claims are based on the number of distinct children, rather than the number of claims. Furthermore, we computed the average quantity of both any drug and PGx drugs that were claimed by each child. Additionally, PGx drugs and associated genes were ranked based on the number of children with claims.

All analyses were performed using SAS 9.4 (SAS Institute Inc., Cary, NC, USA).

### Ethics approval

Ethics approval and informed consent was not necessary according to article 22 of the Swiss Federal law on data protection, as the study was retrospective and used anonymized data [39].

## Results

### Study population

A total of 309 123 children were insured by Helsana between 2017 and 2021, with 159 172 (51.5%) registered throughout the entire five-year period. Of the children present during the entire five-year period, 48.6% were female, 5.4% < 1 year old, 11.1% 1 year old, 28.3% 2–4 years old, 57.9% 5–10 years old, and 60.1% 11–17 years old at least once during the five-year period. Throughout the five-year period, 95.3% of children claimed at least one drug, 95.8% in females and 94.8% in males. Drug prevalence ranged from 88.1% in adolescents to 98.3% in toddlers and was similar in boys and girls. On average, children claimed 10.7 ± 8.0 drugs during the 5-year study period, 10.7 ± 8.1 in girls and 10.6 ± 8.0 in boys (Table 2). The characteristics of the study population in each individual year (2017, 2018, 2019, 2020, and 2021) are shown in the appendix (Appendix Table 1).

**Table 2:** Characteristics of the study population of the five-year period.

	All children *N* = 159 172	Age groups				
		<1 year	1 year	2–4 years	5–10 years	11–17 years
		*N* = 8 607	*N* = 17 602	*N* = 45 104	*N* = 92 111	*N* = 95 686
Children	Number of children [N, %]					
Male	81 850, 51.4	4 459, 51.8	9 025, 51.3	23 141, 51.3	47 404, 51.5	49 240, 51.5
Female	77 322, 48.6	4 148, 48.2	8 577, 48.7	21 963, 48.7	44 707, 48.5	46 446, 48.5
Children with drug claims						
Any drug	151 632, 95.3	7 790, 90.5	17 296, 98.3	41 764, 92.6	81 906, 88.9	84 324, 88.1
≥1 PGx drugs	105 192, 66.1	1 064, 12.4	8 516, 48.4	27 328, 60.6	50 153, 54.4	48 543, 50.7
≥2 PGx drugs	35 396, 22.2	72, 0.8	911, 5.2	5 920, 13.1	12 991, 14.1	14 894, 15.6
≥3 PGx drugs	9 231, 5.8	7, 0.1	74, 0.4	1 009, 2.2	2 803, 3.0	4 195, 4.4
≥4 PGx drugs	2 319, 1.5	1, <0.1	8, <0.1	147, 0.3	672, 0.7	1 181, 1.2
≥5 PGx drugs	487, 0.3	0, 0.0	0, 0.0	19, <0.1	110, 0.1	290, 0.3
≥10 PGx drugs	3, <0.1	0, 0.0	0, 0.0	0, 0.0	0, 0.0	3, <0.1
≥1 syst PGx drugs	100 249, 63.0	926, 10.8	8 159, 46.4	26 255, 58.2	46 881, 50.9	46 002, 48.1
≥1 PGx drug excl. ibuprofen	44 815, 28.2	296, 3.4	1 506, 8.6	7 847, 17.4	17 884, 19.4	20 863, 21.8
≥1 syst PGx drug excl. ibuprofen	28 976, 18.2	125, 1.5	708, 4.0	4 597, 10.2	9 391, 10.2	15 707, 16.4
≥2 syst PGx drug excl. ibuprofen	6 438, 4.0	4, <0.1	49, 0.3	642, 1.4	1 793, 1.9	3 772, 3.9
≥3 syst PGx drug excl. ibuprofen	1 604, 1.0	1, <0.1	2, <0.1	110, 0.2	409, 0.4	1 008, 1.1
Mean number of (PGx) drugs per child ± sd						
Drugs	10.7 ± 8.0	4.9 ± 3.3	8.2 ± 4.2	8.0 ± 6.3	6.7 ± 6.2	7.6 ± 7.4
PGx drugs	1.0 ± 0.9	0.1 ± 0.4	0.5 ± 0.6	0.8 ± 0.7	0.7 ± 0.8	0.7 ± 0.9

*N* number of children, *sd* standard deviation, *PGx* pharmacogenetic, *syst* systemically administered, *excl* excluding, *%* percentage of all children of an age group.

### PGx drugs

**Fig. 1:**
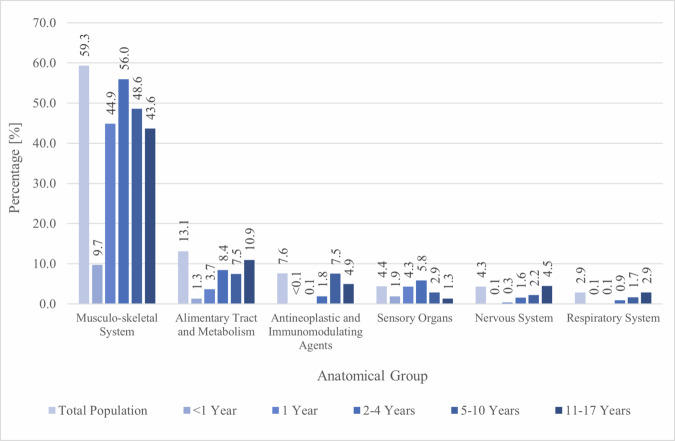
Percentage of children with PGx drug claims stratified by anatomical groups and age groups. The anatomical groups are ranked by the proportion of children who claimed a PGx drug in the total population. Anatomical groups excluded, because < 1.0%: dermatologicals, antiinfectives for systemic use, cardiovascular system, blood and blood forming organs, antiparasitic products, insecticides and repellents, various, genito urinary system and sex hormones, systemic hormonal preparations, excl. sex hormones and insulins. PGx pharmacogenetic, % percentage of the total number of children per age group.

105 192 of the 159 172 children, who were registered for the full five-year study period, claimed at least one PGx drug (66.1%) during the study period. More than two thirds (69.4%) of the children having at least one drug claim for any drug claimed one of the 82 PGx drugs included in our study. Only 6% of all children claimed ≥ 3 PGx drugs. Boys more frequently claimed PGx drugs in children up to 10 years old, while girls older than 10 years more frequently claimed PGx drugs. When excluding ibuprofen and locally administered PGx drugs 18.2% of children claimed PGx drugs during the five-year period. The total number of children claiming PGx drugs ranged from 94 092 for systemically administered ibuprofen to one each in atazanavir, fluvastatin, methylene blue, paromomycin, phenytoin, primaquine, and tenoxicam (Table 3 and Appendix Table 2). The top three claimed PGx drugs regardless of sex were systemically administered ibuprofen, ondansetron, and locally administered fluorouracil with 94 092, 13 269, and 11 982 users, respectively (Table 3). 25 out of the 82 drugs were not claimed during the five-year study period, because 20 PGx drugs were either not approved for use in children in Switzerland or not approved at all. Furthermore, amikacin, doxepin, flurbiprofen, gentamicin, and ibuprofen were not administered locally at any point during the five-year-period. However, only flurbiprofen and ibuprofen were approved for local administration in adolescents in Switzerland during the study period. Grouping the drugs by anatomical group revealed that the majority of children claimed PGx drugs from the musculo-skeletal system, followed by those targeting the alimentary tract and metabolism, and antineoplastic and immunomodulating agents (Fig. 1). After excluding locally administered drugs, we found that most children regardless of sex claimed PGx drugs from the musculo-skeletal system, the alimentary tract and metabolism, and the nervous system (Appendix Figure 1). The distribution after additionally excluding ibuprofen is provided in the appendix (Appendix Figure 2).

**Table 3:** Top 15 PGx drugs in the five-year period stratified by age groups.

The drugs are ranked by the number of exposed children of the total population. In blue: rank 1, in green: rank 2, and in orange: rank 3.

*N* number of children, *%* percentage of all children present in the respective age group, *PGx* pharmacogenetic, *syst* systemically administered, *loc* locally administered.

Comparisons across the different age groups revealed that the PGx drug prevalence ranged from 12.4% in infants to 60.6% in children 2–4 years old. Based on the number of subjects claiming PGx drugs in the respective age groups, systemic ibuprofen ranked first across all age groups in boys and girls, while second and third rank varied in the different age groups. Locally administered tobramycin was more frequently used by younger children, while locally administered fluorouracil was primarily used by children aged 5 and over. Ondansetron was frequently used across all age groups. Gender differences in PGx drug utilization were observed for certain drugs. For instance, girls were found to use sertraline (0.7 vs. 0.3%), escitalopram (0.4 vs. 0.2%), and nitrofurantoin (0.4 vs. <0.1%) more frequently, while boys were found to use risperidone (0.2 vs. 0.6%) and atomoxetine (0.1 vs. 0.3%) more frequently during the five-year period. The number of users during the whole five-year period, as well as annual PGx drug prevalences stratified by age groups, are available in the appendix (Appendix Table 2 to Appendix Table 8).

### PGx genes

The ranking of genes associated with PGx drugs showed that CYP2C9, CYP2D6, and DPYD were the genes most frequently associated with the drugs used by the whole study population (Table 4), but the frequency of exposure and the rank within the different age groups varied across childhood and adolescence. During the study period, no children claimed drugs associated with CFTR and CYP4F2.

**Table 4:** Genes associated with PGx drugs in the five-year period.

Genes are ranked by highest number of children in the total population with claims for associated drugs. In blue: rank 1, in green: rank 2, and in orange: rank 3.

Percentages can add up to over 100%, as some children were exposed to more than one PGx drug.

*PGx* pharmacogenetic.

**Fig. 2:**
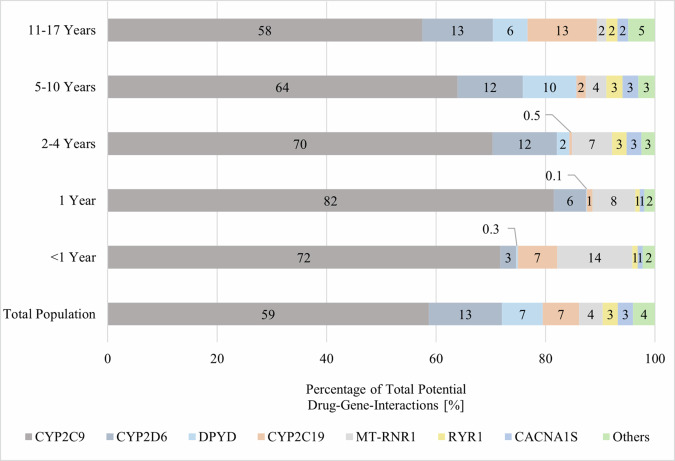
Proportion of all potential drug-gene interactions, stratified by age groups. Ranked by the total population. Others include: CYP3A4, CYP3A5, CYP2B6, G6PD, TPMT, NUDT15, HLA-B, SLCO1B1, HLA-A, ABCG2, SLC28A3, RARG, UGT1A6, VKORC1, UGT1A1, CFTR, CYP4F2.

96.1% of all potential DGIs found in the total population were attributed to seven genes: CYP2C9, CYP2D6, DPYD, CYP2C19, MT-RNR1, CACNA1S, and RYR1 (Fig. 2). When PGx drugs administered locally were excluded, five genes were found to cause 98.7% of all potential DGIs in the total population. These genes were: CYP2C9, CYP2D6, CYP2C19, RYR1, and CACNA1S (Appendix Figure 3). After additionally excluding ibuprofen the following five genes CYP2D6, CYP2C19, RYR1, CACNA1S, and CYP2B6 were associated with 97.0% of all potential DGIs in the total population (Appendix Figure 4). The proportion of CYP2D6 increased with the age of the children and had a greater impact on older children. In contrast, MT-RNR1 held the largest proportion of all potential DGIs in infants. CYP2C19 was responsible for a significant proportion of all potential DGIs in infants.

## Discussion

This study determined prescription patterns of PGx drugs and assessed the potential of PGx testing in paediatrics for the first time in Switzerland. During the five-year study period, 66.1% of children claimed at least one PGx drug, which is high compared to PGx drug prevalences reported in previous studies [30, 31, 33, 40]. Comparisons across different studies are complicated by various factors. For example, PGx dosing guidelines, such as CPIC or DPWG guidelines, are regularly updated. Additionally, different countries have different drugs on the market and use different criteria to define paediatric age groups. Furthermore, studies covered different study periods. The CPIC guideline for ibuprofen, which was a highly prevalent drug in our study, has only been published in 2020; for that reason, it was not included in some of the previous studies [23, 28–30, 40]. Ramsey et al. reported an annual PGx drug exposure prevalence of 8.0–10.6% in children in the USA [30]. Their investigation was, however, limited to systemically applied CPIC level A drugs in persons < 21 years. Ibuprofen was not classified as a CPIC level A drug at the time of their study, which explains at least in part the large difference in PGx drug prevalence, as ibuprofen was the drug with the highest prevalence in our study [30]. Samwald et al. estimated that between 11.2 and 14.0% of children aged 0–13 years received at least one PGx drug during a four-year span. They did also not include ibuprofen as a PGx drug and excluded locally administered PGx drugs [40]. Liu et al. found a 29% prevalence of PGx drug exposure in children living in the USA aged 0–17 years within a three-year period [31]. Only Liu et al. included ibuprofen as a PGx drug [31]. To increase the validity of comparisons with previous studies, we did additional analyses in which we excluded ibuprofen and locally administered drugs. We then observed a five-year PGx drug prevalence of 18.2% in our study population.

When we focus on claims of at least two or three systemic PGx drugs (excluding ibuprofen), the prevalence was 4.0 and 1.0%, respectively, amongst the paediatric population, which is also higher than in previous studies from other countries. Samwald et al. reported that exposure to at least two PGx drugs was observed in 1.1–1.8% of children, while exposure to at least three PGx drugs was seen in 0.2–0.5% of children [40]. Ramsey et al. observed an increase in exposure to at least two PGx drugs from 1.5% in 2011 to 2.2% in 2017 [30].

In our study we observed a high ibuprofen prevalence of 59.1%. Ibuprofen is used in children to treat pain and fever [41]. The only other study to include ibuprofen as a PGx drug was conducted by Liu et al., using claims data from 2017–2019 in the USA. In children aged 0–17 years, the three-year prevalence of ibuprofen was 2.9% [31]. The prevalence for ibuprofen in our study was much higher. Non-PGx studies from the USA, Finland, and the Netherlands also found lower prevalences for ibuprofen and NSAIDs [42–44]. Differences in prevalences can have many causes: they are influenced by how the health system is organised, which reimbursement is offered by insurance companies, who is insured, and what drugs in which pharmaceutical forms are available for children OTC. In Switzerland, ibuprofen can be bought OTC and is therefore likely to even be underestimated in our study [45].

The antiemetic drug ondansetron (8.3%) was also quite prevalent in our study population, an observation which corresponds to results from other studies [28–32]. Besides its approved indications, ondansetron is also used off-label for the treatment of acute gastroenteritis [46, 47]. Additionally, it is the sole antiemetic approved for treating postoperative and radiation-induced vomiting in Switzerland from the first month of life [45].

Compared to the high prevalence of depression in adolescents (33%) [48], the number of claims of PGx antidepressants is low (escitalopram = 0.5%, sertraline = 0.8%). One explanation for this result is that, according to current guidelines, sertraline is the second-line treatment for unipolar depression in children and adolescents. The first choice is fluoxetine, which is not a PGx antidepressant and was therefore not investigated further in our study [49]. Tricyclic antidepressants, which are also PGx drugs, have shown no benefit in treating depression in children and are therefore unlikely to be used in our population [50].

In contrast to other studies, we included locally applied drugs as PGx drugs [30, 31, 40], because there are some reports on toxicity with locally applied PGx drugs [51–54]. In our study, three locally administered PGx drugs (fluorouracil, tacrolimus, and tobramycin) were among the top PGx drugs. The prevalence of local and systemic PGx drugs varied by age groups. Locally administered fluorouracil was mainly used in children aged 5–10 years. As locally administered fluorouracil can be used to treat warts, which are common in this age group [55], we assume that this explains why it is more prevalent in this than in other age groups in our study. There is only one locally applied product containing fluorouracil approved for use in children in Switzerland. It is a solution containing 5% fluorouracil [45]. According to the medicinal product information, the solution is applied 2–3 times a day for an average of 6 weeks. The solution should be applied to a maximum of 25 cm^2^ of body surface area, resulting in the application of 1 mg of fluorouracil [45]. Cohen et al. described a case of an adult male using 5% fluorouracil cream who developed severe neutropenia after 14 applications of the cream over 11 days [51]. The doses used in this case and in children in Switzerland overlap. We therefore think that toxic reactions to locally applied fluorouracil may indeed be relevant in children. Topical tobramycin was more prevalent in children under 4 years of age than older children. This was also observed in a French study, where children under 6 years were more likely to receive ophthalmic anti-infectives than older children [56]. In children aged 0–10 years, omeprazole was used more often than pantoprazole. However, pantoprazole was more commonly used in adolescents. In Switzerland, omeprazole is approved for infants, while pantoprazole is approved from the age of 12 years on [45]. Both substances can be used off-label from the age of one month [57]. Omeprazole is available in a multiple-unit-pellet-system (MUPS) formulation, making it easy to suspend for use in children [45]. Pantoprazole also has a granule formulation, but unlike MUPS-omeprazole, it is not reimbursed by health insurance [45, 58]. These differences could explain why omeprazole was used more often in younger children.

Out of 24 genes associated with the 82 studied drugs, CYP2C9 ranked first when assessing the number of children who claimed a drug associated with this gene, followed by CYP2D6 and DPYD. This is not surprising as ibuprofen, the most prevalent drug in our study population, is associated with CYP2C9 [59]. In contrast to other studies, we observed no drugs claims for drugs associated with CFTR and CYP4F2. Ivacaftor, which is associated with CFTR, was not claimed in the paediatric population, although it is available in Switzerland. Warfarin, which is associated with CYP4F2, is not approved in Switzerland and has therefore never been claimed [45]. For a better comparison with previous studies, we again excluded locally administered drugs and ibuprofen from the analysis. CYP2D6, CYP2C19, and CACNA1S/ RYR1 ranked first, second, and third, which corresponds to the results by Ramsey et al. [30]. The genes CYP2C9, CYP2D6, DPYD, MT-RNR1, CYP2C19, CACNA1S, and RYR1 were responsible for over 96% of all potential DGIs. Studies from the Netherlands, the UK, and Switzerland came to similar conclusions, although they did not focus on the paediatric population [33, 60, 61]. These three studies found that a higher percentage of DGIs were caused by SLCO1B1 [33, 60, 61]. SLCO1B1 is most commonly associated with statins, which are rarely used in children [62].

Our study has a number of strengths and limitations. A notable strength of this study lies in its use of multiple sources of recommendations for the definition of PGx drugs. Furthermore, solely PGx guidelines with recommendations were included, to ensure that the study is relevant to the current clinical decision-making process. We included medications applied locally and systemically, and we present results for both, which - to our knowledge - no other study has done yet. The classification of PGx drugs as either locally or systemically administered was challenging, due to the absence of differentiation in some ATC codes. Another challenge was the fact that age groups are defined differently in various countries, and they do not have the same duration. Such disparities in definitions make it difficult to compare our results with those of others. Our study is based on claims data and is therefore subject to the limitations of those data. We did not have information regarding the utilisation of OTC medication. Our research may have underestimated the prevalence of three PGx drugs (codeine and flurbiprofen both approved for use from the age of 12 years, and ibuprofen approved for use from the age of 6 months) that are available OTC for children in Switzerland. Additionally, we lacked information on drugs used in hospitals, as these are billed at a case rate except for highly expensive drugs [63]. Hence, our study may have underestimated medications frequently used in hospitals. Furthermore, the used claims database does not provide diagnoses; thus, it is impossible to determine the indication of the administered drugs. Importantly, the database lacks any genetic data of individuals. Consequently, it is impossible to determine the frequency of actionable DGIs and the impact of PGx testing on children. Only about 50% of the children registered during the five-year period were continuously insured. As Swiss insurances are private companies, the fees for standard insurance fluctuate annually. In Switzerland it is mandatory to have basic health insurance, but Swiss people are free to choose and to switch between insurances on an annual basis [64]. As a result, some individuals do indeed change their insurance every year, resulting in this fluctuation. Everybody is required to pay a (small) yearly amount before they can be reimbursed. This can lead to some drugs that have not been billed to the health insurance and may have been missed. As out-of-pocket payment has a greater impact on acute than on chronic medication, it is possible that some drug claims have been omitted. This could have resulted in a minor underestimation of acute medication in our findings. Furthermore, it should be noted that health care claims data cannot determine if the medication was actually taken by the individuals. Therefore, it is probable that the actual exposure to PGx drugs may be lower if the medication was not taken.

To our knowledge, this study is the first to evaluate PGx drug claims among children residing in Switzerland. Our study indicates a high utilisation of PGx drugs among children in Switzerland, with 66.1% claiming PGx drugs during a five-year period. Among PGx drugs, the NSAID ibuprofen, the antiemetic ondansetron and locally administered fluorouracil had the highest number of users. We identified CYP2C9, CYP2D6, DPYD, and CYP2C19 as relevant pharmacogenes in paediatrics. As the utilisation of PGx drugs is frequent and few genes are responsible for a high amount of potential DGIs, we propose to rather use a preemptive testing panel in paediatrics than single gene testing. However, further studies are required to evaluate the clinical impact of PGx in the paediatric population.

## Supplementary information

Supplementary Figures

Supplementary Tables

## Data Availability

The datasets generated and/or analysed during the current study are not publicly available due to confidentiality requirements issued by Helsana. Analysis codes and datasets can be made available by the corresponding author (s.allemann@unibas.ch) upon reasonable request and with permission of Helsana.

## Code availability

Analysis codes can be made available by the corresponding author (s.allemann@unibas.ch) upon reasonable request and with permission of Helsana.
