# Supplementary material for: Utilisation of drugs with pharmacogenetic recommendations in children in Switzerland
Source: Pharmacogenomics J. 2025 Jul 11;25(4):19. doi: 10.1038/s41397-025-00378-x (PMC12254038; doi:10.1038/s41397-025-00378-x)
Supplement: Supplementary file 1 — Supplementary Figures [file 41397_2025_378_MOESM1_ESM.pdf]

Appendix

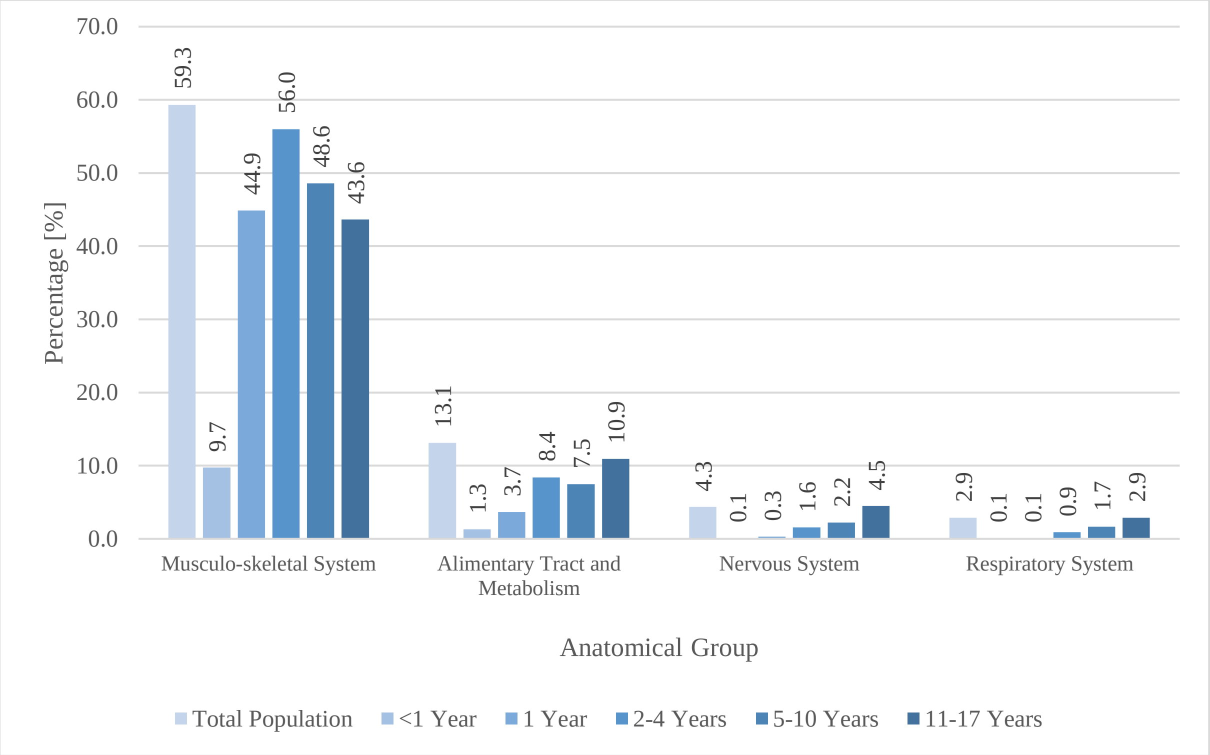

Appendix Figure 1 Percentage of children with systemically administered PGx drug claims stratified by anatomical groups and age groups. The anatomical groups are ranked by the proportion of children exposed in the total population.

Notes: Anatomical groups excluded, because <1.0%: antiinfectives for systemic use, antineoplastic and immunomodulating agents, cardiovascular system, blood and blood forming organs, antiparasitic products, insecticides and repellents, various, dermatologicals, genito urinary system and sex hormones, systemic hormonal preparations, excl. Sex hormones and insulins, sensory organs.

Abbreviations: PGx: pharmacogenetic, %: percentage of the total number of children per age group.

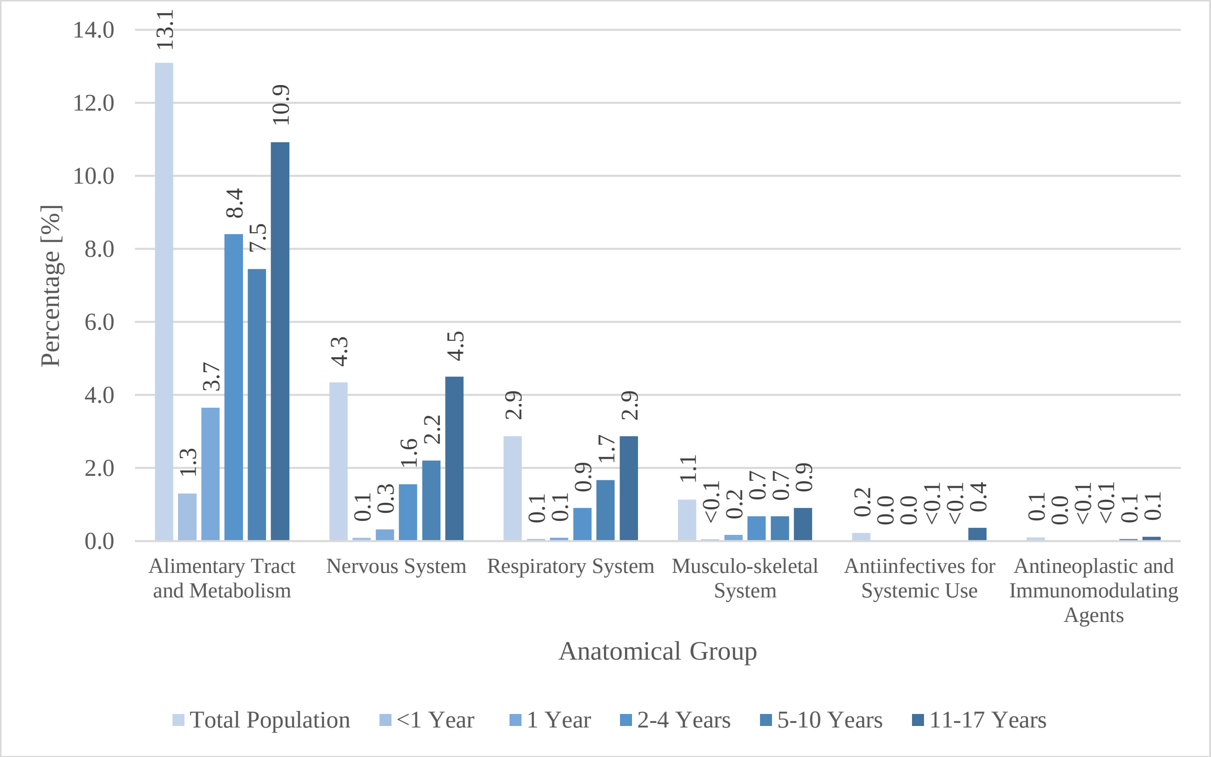

Appendix Figure 2 Percentage of children with systemically administered PGx drug claims excluding ibuprofen stratified by anatomical groups and age groups. The anatomical groups are ranked by the proportion of children exposed in the total population.

Notes: Anatomical groups excluded, because <1.0%: antiinfectives for systemic use, antineoplastic and immunomodulating agents, cardiovascular system, blood and blood forming organs, antiparasitic products, insecticides and repellents, various, dermatologicals, genito urinary system and sex hormones, systemic hormonal preparations, excl. Sex hormones and insulins, sensory organs.

Abbreviations: PGx: pharmacogenetic, %: percentage of the total number of children per age group.

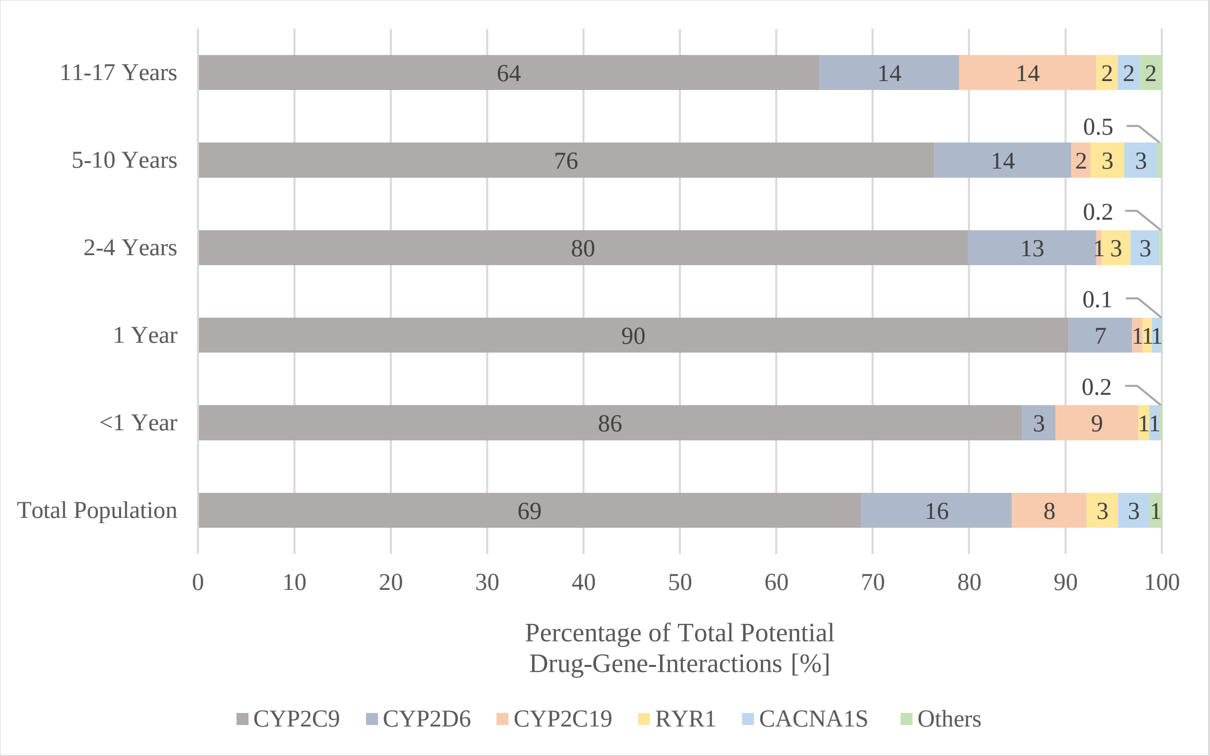

Appendix Figure 3 Proportion of all potential drug-gene interactions by systemically applied PGx drugs, stratified by age groups. Ranked by the total population.

Others include: CYP2B6, G6PD, TPMT, NUDT15, HLA-B, SLCO1B1, HLA-A, ABCG2, SLC28A3, RARG, UGT1A6, CYP3A4, CYP3A5, DPYD, MT-RNR1, VKORC1, UGT1A1, CFTR, CYP4F2.

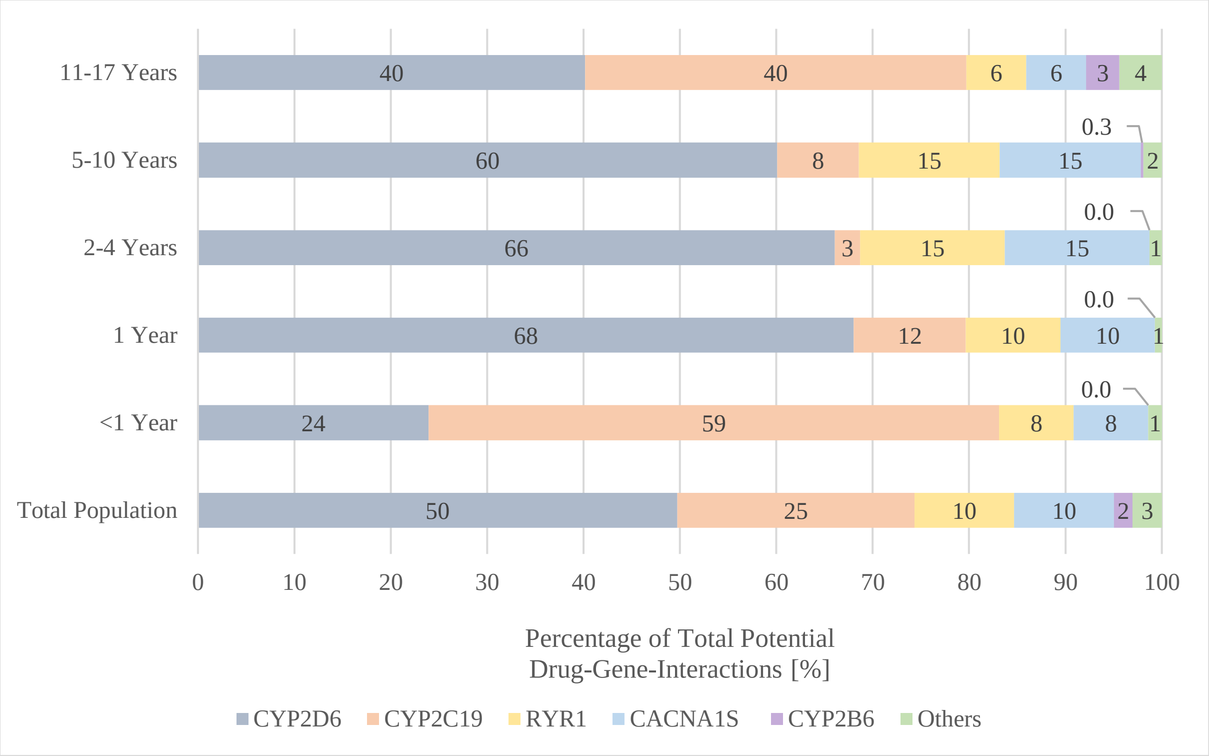

Appendix Figure 4 Proportion of all potential drug-gene interactions by systemically applied PGx drugs excluding ibuprofen, stratified by age groups. Ranked by the total population.

Others include: CYP2C9, G6PD, TPMT, NUDT15, HLA-B, SLCO1B1, HLA-A, ABCG2, SLC28A3, RARG, UGT1A6, CYP3A4, CYP3A5, DPYD, MT-RNR1, VKORC1, UGT1A1, CFTR, CYP4F2.
